# Effect of acupuncture and auricular acupressure on smoking cessation: Protocol of a systematic review and Bayesian network meta-analysis

**DOI:** 10.1097/MD.0000000000020295

**Published:** 2020-05-29

**Authors:** Runjing Dai, Jie Zhang, Hailiang Zhang, Na Zhao, Fujian Song, Jingchun Fan

**Affiliations:** a School of Public Health, Center for Evidence-Based Medicine; b The Graduate School, Affiliated Hospital of Gansu University of Chinese Medicine, Lanzhou City, Gansu Province, P.R. China; c Norwich Medical School, University of East Anglia, Norwich, Norfolk, UK.

**Keywords:** acupuncture, auricular acupressure, network meta-analysis, nicotine replacement, smoking cessation

## Abstract

**Background:**

Tobacco epidemic remains a major challenge to public health, with >7 million deaths attributable to tobacco smoking p.a. Quitting smoking is a proven way of reducing the harm of smoking. Nicotine replacement therapy (NRT), auricular acupressure and acupuncture are used for quit smoking, but it remains to be explored which is relatively more effective. Furthermore, a Bayesian network meta-analysis will be applied to determine the relative effects and/or safety of different smoking cessation treatments.

**Methods/design:**

A literature search for randomized controlled trials (RCTs) will be performed in five electronic databases from inception to December 2019, including PubMed, the Cochrane library, EMBASE, Web of Science, and Chinese Biomedical Database (SinoMed). Cochrane Collaboration quality assessment tool will be used for the risk of bias assessment. A Bayesian network meta-analysis will be performed using WinBUGS 1.4.3, and Stata 14 will be applied to draw the network diagram, while RevMan 5.3.5 will be used to produce funnel plot for assessing the risk of publication bias. Recommended rating, development and grade methodology will also be utilized to assess the quality of evidence.

**Results:**

We will evaluate the effect of different smoking cessation treatments (e.g., acupuncture, auricular acupressure, and NRT) by directly traditional meta-analysis and indirectly Bayesian network meta-analysis.

**Conclusion:**

Our study will provide smokers with the available evidence on the efficacy and safety of quitting regimens.

## 1 Introduction

Tobacco is harmful to human being in any form, including smoking and smokeless products with fatal health problems.^[1]^ The World Health Organization (WHO) reported that tobacco epidemic remains a major public health threat ever faced around the world with mortality >8 million people, particularly >7 million were tobacco directly related deaths.^[2]^ It is well known that tobacco use is one of the major risk factors contributing to the development of many chronic diseases, especially in the respiratory and cardiovascular systems.^[3]^ Therefore quit smoking probably is the only proven way to reduce such harmful consequence(s).^[4]^

The most common ways to quit smoking include medication such as Varenicline, nicotine replacement therapy (NRT), hypnosis, education, and behavioral intervention.^[5–9]^ In addition, some traditional Chinese medicine therapies such as acupuncture and auricular acupressure are also reported.^[10,11]^ It has been demonstrated that NRT, acupuncture and auricular acupressure have certain effect(s) on smoking cessation.^[8,10,12]^ The results showed that acupuncture combined with auricular acupressure and NRT combined with psychotherapy have better effect on smoking cessation than any of the single one.^[8,10]^ Interestingly it is acknowledged that age and sex are the influencing the effect of smoking cessation.^[10,12]^ However, the conclusion is not to solid due to the insufficient experimental design or sample size. A number of meta-analyses on smoking cessation have been published to assess the effectiveness of the different approaches in smoking cessation. The safety and effectiveness in smoking cessation have been determined in single and/or combined therapy, including acupuncture, acupressure, laser therapy, and electrical stimulation, as well as the comparison of sham therapy or other interventions.^[13,14]^ Furthermore, auricular acupuncture, auricular acupoint pressure, auricular therapy, and acupoint stimulation are also evaluated, relating to daily cigarette consumption.^[15,16]^ Most of the meta-analysis published are conducted a direct comparison of the various interventions, rather than an indirect comparison of the interventions, and do not give a clear effect on smoking cessation programs. Thus, we will explore the relative effects and/or safety of different smoking cessation treatments through the Bayesian network meta-analysis.

## 2 Methods/design

### 2.1 Research objectives

The review will be conducted to evaluate the effect of NRT, auricular acupressure alone, acupuncture, acupuncture combined with auricular acupressure, and sham acupoint therapy on smoking cessation, to determine the relative effects and/or safety way to quit smoking.

### 2.2 Inclusion criteria

#### 2.2.1 Types of studies

We will include only randomized controlled trials (RCTs) with acupuncture, NRT, auricular acupressure, and other interventions.

#### 2.2.2 Types of participants

(1) The tobacco smokers who wish to stop smoking
(2) Smokers receiving acupuncture, ear acupuncture and body acupuncture, auricular acupressure alone, or acupuncture combined with auricular acupressure.

#### 2.2.3 Types of interventions

The eligible interventions include acupuncture, ear acupuncture and body acupuncture, auricular acupressure alone, or acupuncture combined with auricular acupressure.

#### 2.2.4 Types of comparators

The control interventions will be included sham acupuncture, sham auricular acupressure, NRT or auricular acupressure alone.

#### 2.2.5 Types of outcomes

Our eligibility criteria will include all outcomes that reported in the included study.

### 2.3 Exclusion criteria

The exclusion criteria include:

1. Quitters who receive any combination of two or more therapies other than auricular acupressure combined with acupuncture.
2. Any acupuncture with electric or laser stimulation.
3. Case reports, reviews, abstracts, experimental studies, mechanism discussions, experience summary, and other research types of literature.
4. Repeatedly checked or published literature.
5. Incomplete data or information that does not indicate the end result and cannot be included.

### 2.4 Information sources and search strategy

A literature search for RCTs will be performed in five electronic databases from inception to December 2019, including one Chinese and four English databases: Chinese Biomedical Database (SinoMed), PubMed, the Cochrane Library, EMBASE, and Web of Science. In order to find more relevant papers, we will conduct forward and backward citation screening through the citation and bibliography of systematic review. Search word for smoking cessation, nicotine replacement, acupuncture, and auricular acupressure. Multiple synonyms for each word will be incorporated into the search. Search strategy of PubMed is provided in Table 1.

**Table 1:**
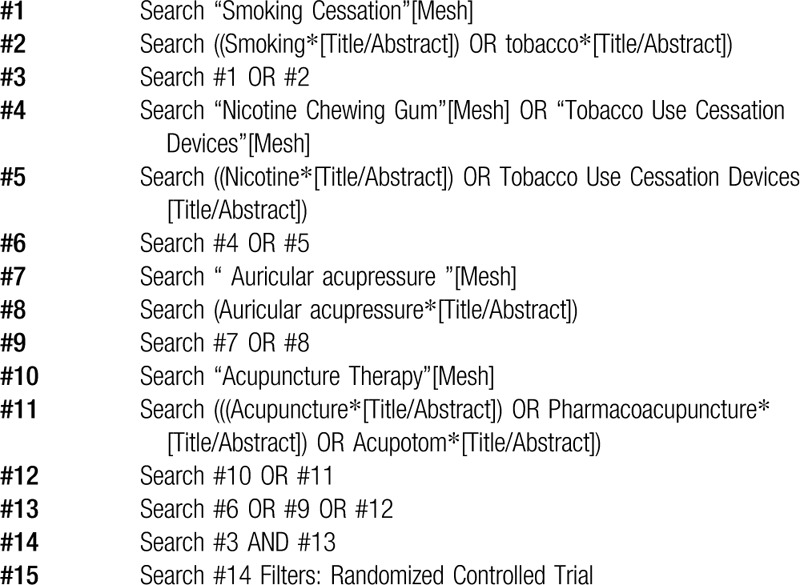
Search strategy (PubMed).

### 2.5 Date management

The document search results will be uploaded to the document management software (Endnote X9). Before the formal selection of literature, we will conduct two inspections after the literature is exported and the duplicate literature is excluded. The software will record our screening process.

### 2.6 Study selection

**Figure 1:**
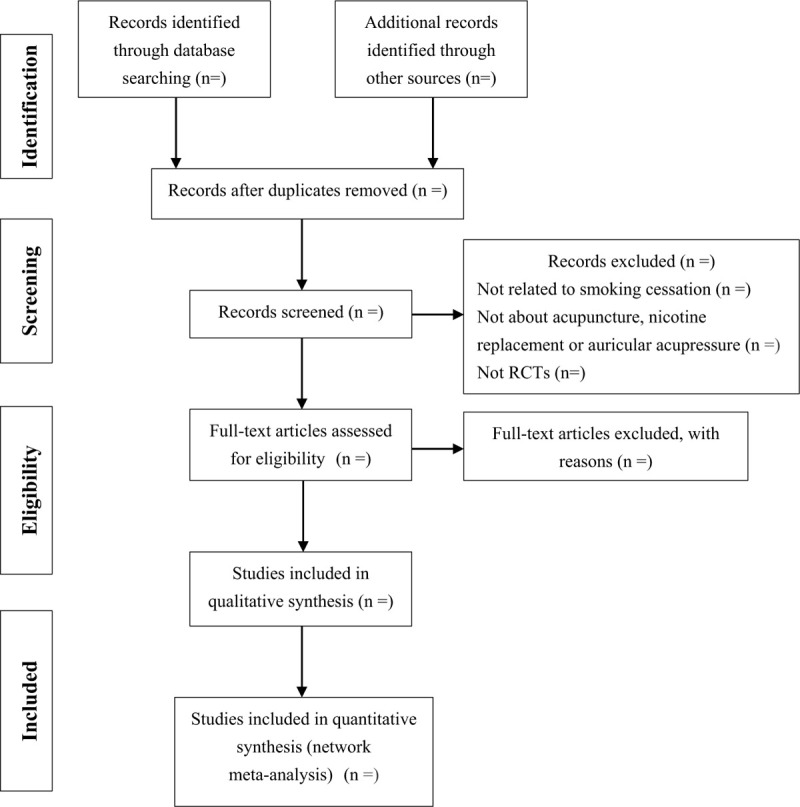
Preferred reporting items for systematic review and Bayesian network meta-analysis.

Researchers will import the literature retrieved to the Endnote X9 and eliminate the duplicate data. Then two reviewers will select the articles independently based on the titles/abstracts and full texts. A final judgment is made after discussion with the third senior researcher in case of disagreement. If the study data is repeated, only the studies with large sample size and long follow-up time will be included.^[17]^ The flow chart of study selection is displayed in Figure 1.

### 2.7 Data extraction

Data will be entered into a systematic coding form that included first author, year of publication, detailed questions on interventions, methods, and outcomes. When multiple treatment durations are reported, data for each time period will be recorded.

### 2.8 Outcomes

The main outcome measures will be abstinence rate, including abstinence rate at the end of treatment and abstinence rate at the end of follow-up from 1 to 6 months. Sustained abstinence rate will be used as the preferable outcome. If the sustained abstinence outcome is not available, point abstinence rate will also be acceptable. Abstinence rates can be measured by the expiration test for carbon monoxide content or cotinine in the urine or cigarette withdrawal symptom scores or self-developed criteria.

### 2.9 Risk of bias assessment

The methodological quality of each included study will be assessed using the Cochrane Collaboration quality assessment tool by two independent reviewers. The assessment tool includes the following criteria: random sequence generation, allocation concealment, blinding of participants and personnel, blinding of the results assessment, incomplete data of the results, selective reporting, and other sources of bias.^[18]^ Disagreement will be decided by discussion with a third senior investigator.

### 2.10 Date synthesis and analysis

Direct comparison and indirect comparison will be performed in this study. WinBUGS (V.1.4.3; MRC Biostatistics Unit, Cambridge University, UK) will be used for the Bayesian network meta-analysis, and Stata (V.14; StataCorp) will be used to draw the network diagram, while Review Manager Software (V.5.3.5; RevMan) will be used to produce funnel plot for assessing the risk of publication bias. The visual assessment method to test funnel plot asymmetry will be applied to assess small size effects.^[19]^ The odds ratio (OR) for dichotomous data, weighted mean difference (WMD) for continuous data, and 95% credible intervals (95%CI) will be used to estimate the network-analysis. The consistency model analysis of the main outcome indexes and the probability ranking of the best treatment measures will be carried out, and the node model analysis of the network graph with closed rings will be carried out to evaluate the consistency. Potential Scale Reduction Factors (PSRF) will be used to evaluate the model convergence. The closer PSRF will be to 1, the better the model convergence is. A two-tailed value of *P* ≤ .05 will be considered to indicate statistical significance. We will use the *χ*^2^ test to estimate the presence of statistical heterogeneity with threshold as *P* ≤ .05. The *I*^2^ test will be used to estimate the degree of heterogeneity as truncating *I*^2^ ≥ 50% as considerable heterogeneity. The sensitivity analysis will be conducted if the heterogeneity is statistically significant. The purpose of these studies was to explore whether this might have an impact on our results.

#### 2.10.1 Confidence in cumulative estimate

We will use the recommended rating, development, and rating methods to assess the quality of direct and indirect evidence.^[20]^ The quality of evidence will be graded as high, moderate or low. The above work will be performed by two independent reviewers. If there are different opinions, the decision will be made after consultation with the third investigator.

#### 2.10.2 Assessment of publication bias

Bergg's and Egger's funnel plot methods will be used to help distinguish the asymmetry due to publication bias.^[21,22]^

### 2.11 Ethics and dissemination

There is no research ethical issue because this is a systematic review of published literature. The results of this study will be published in a peer-reviewed journal.

## 3 Discussion

The purpose of this study was to conduct a network meta-analysis on the effects of NRT, auricular acupressure alone, acupuncture, acupuncture combined with auricular acupressure, and sham acupoint therapy on smoking cessation, and to determine the relative effects and/or safety way to quit smoking. We believe that our study will provide smokers with the available evidence on the efficacy and safety of quitting regimens, which will hopefully contribute to future clinical trials and study design. In this study, we will only include RCTs. Of course, in order to ensure that the RCTs literature included is comprehensive, we will track and retrieve relevant references for published systematic evaluations and meta-analyses. In addition, although there are a large number of literatures on the effect of acupuncture and other traditional Chinese medicine methods on smoking cessation, the number of literatures on RCTs may also be relatively small and the literature quality is not high, which may affect the results of this study.

## Acknowledgments

We are grateful for the support we received from the Evidence-based Medicine Center of Lanzhou University, Dr. Jinhui Tian, while developing our search strategies and constructive comments during the review process.

## Author contributions

All listed authors have contributed and will continue to contribute meaningfully to the protocol and proposed review. RJD and JZ are guarantors and contributed equally to this work. RJD and JCF planned and designed the research. FJS tested the feasibility of the study. RJD, JZ, HLZ and NZ developed the search strategy. RJD and JZ wrote the manuscript. JCF and FJS provided methodological advice, polished and revised the manuscript. JCF will be the third senior reviewer that will help resolve any discrepancy. All authors approved the final version of the manuscript.
